# Recovery of T‐cell function in healthy dogs following cessation of oral cyclosporine administration

**DOI:** 10.1002/vms3.230

**Published:** 2020-01-08

**Authors:** Lakshmi Narayanan, Charlee Mulligan, Lisa Durso, Brittany Thames, John Thomason, Claire Fellman, Andrew Mackin, Robert Wills, Todd Archer

**Affiliations:** ^1^ Department of Clinical Sciences Mississippi State University College of Veterinary Medicine Mississippi State MS USA; ^2^ Department of Pathobiology and Population Medicine Mississippi State University College of Veterinary Medicine Mississippi State MS USA; ^3^ Present address: Department of Clinical Sciences Tufts University North Grafton MA USA; ^4^ Present address: Big Country Veterinary Clinic Abilene TX USA; ^5^ Present address: Veterinary Emergency Referral Center Pensacola FL USA

**Keywords:** cyclosporine, cytokines, interferon gamma, interleukin 2, T cell

## Abstract

Cyclosporine is a potent immunosuppressive agent used to treat immune‐mediated disorders in dogs. Secondary infections sometimes necessitate withdrawal of cyclosporine, but it is not known how long it takes for the immune system to recover after cessation of cyclosporine. Our goal was to utilize a validated RT‐qPCR assay in dogs to assess recovery time of the T‐cell cytokines IL‐2 and IFN‐γ after discontinuation of cyclosporine. Six healthy dogs were given oral cyclosporine (10 mg/kg every 12 hr) for 1 week, with samples collected for measurement of cytokine gene expression prior to treatment, and on the last day of therapy. Cyclosporine was then discontinued, and samples were collected daily for an additional 7 days. Results revealed that there was a significant difference in cytokine expression when comparing pre‐treatment and immediate post‐treatment values, corresponding to marked suppression of T‐cell function. There was no significant difference between pre‐treatment values for either cytokine when compared with any day during the recovery period. Cytokine expression, evaluated as a percentage of pre‐treatment baseline samples, demonstrated progressing return of T‐cell function after drug cessation, with full recovery seen in all dogs by Day 4 of the recovery period.

## Graphical abstract

Cyclosporine administration in dogs did result in a significant difference in ∆Ct for cytokine expression when comparing pre‐treatment and immediate post‐treatment values, corresponding to marked suppression of T‐cell function. Cytokine expression, evaluated as a percentage of pre‐treatment baseline samples, demonstrated progressing return of T‐cell function after drug cessation, with full recovery by Day 3 of the recovery period.

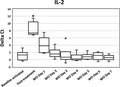

## 1 INTRODUCTION

Dogs are commonly affected by immune‐mediated disorders which can cause significant morbidity and mortality. One of the key therapies for immune‐mediated disorders is immunosuppressive therapy. Cyclosporine is a potent immunosuppressive agent used in veterinary medicine as one option for inducing immune suppression and attempting to achieve disease remission. Cyclosporine functions by forming a complex with cyclophilin A, an immunophilin that is abundant in T cells. The formation of a cyclosporine–cyclophilin complex results in an enhanced affinity of cyclophilin to the intracellular enzyme calcineurin, and this binding inhibits the function of calcineurin and the subsequent activation of the nuclear factor of activated T cells (NFAT) pathway, thus preventing the production of cytokines, such as interleukin‐2 (IL‐2), interleukin‐4 (IL‐4), tumour necrosis factor‐alpha (TNF‐α) and interferon‐gamma (IFN‐γ), thereby blocking activation of T cells and inducing immune suppression. (Archer et al., 2014).

Cyclosporine has been used to try to treat a variety of common inflammatory and immune‐mediated diseases in dogs, such as atopic dermatitis, anal furunculosis, inflammatory bowel disease, immune‐mediated haemolytic anaemia (IMHA), immune‐mediated thrombocytopenia (IMT) and immune‐mediated polyarthritis (IMPA), even though well‐controlled clinical trials have not validated its efficacy for many of these conditions (Archer et al., 2014; Fellman et al., 2011). While cyclosporine is often used to induce immune suppression in these disorders, when the patient's immune system is over suppressed, development of concurrent infections can occur (Archer et al., 2014).

Secondary infections are in fact possible in any patient that is receiving immunosuppressive medications, but are particularly well documented in dogs receiving cyclosporine therapy (Banovic, Kock, Robson, Jacob, & Olivry, 2015; Dedeaux, Grooters, Wakamatsu‐Utsuki, & Toboada, 2018; Galgut, Janardhan, Grondin, Harkin, & Wight‐Carter, 2010; MacNeill, Steeil, Dossin, Hoien‐Dalen, & Maddox, 2010; McAtee et al., 2017; Mohri, Takashima, Yamane, Sato, & Yamane, 2009; Peterson, Torres, Rendahl, & Koch, 2012; Radowicz & Power, 2005; Rhoades, Vernau, Kass, Herrera, & Sykes, 2016; Siak & Burrows, 2013). In a recent review article discussing opportunistic fungal infections in small animals, the authors concluded “Based on published case reports, the authors’ own observations, and a recent large retrospective study of dogs treated for immune‐mediated disease, administration of cyclosporine appears to be a risk factor for the development of opportunistic fungal infections in dogs.” (Dedeaux et al., 2018) In one study evaluating dogs receiving oral cyclosporine therapy at a dose of 5 mg/kg twice daily, 20% developed secondary infections, including one dog that developed generalized demodicosis despite having the lowest trough cyclosporine blood concentration of all dogs receiving cyclosporine (Rhoades et al., 2016). In another retrospective study of dogs treated for immune‐mediated disease, 13% of dogs treated developed an opportunistic invasive fungal infection, with an average cyclosporine dosage of 11.5 mg/kg/day at the time of diagnosis of infection (McAtee et al., 2017). Furthermore, in a study evaluating the development of urinary tract infections in dogs receiving cyclosporine, 30% of cyclosporine‐treated dogs had at least one positive urine culture (Peterson et al., 2012). Previously published results document some dogs developing significant suppression of T‐cell function suppression when administered the relatively low cyclosporine dosage approved for treatment of atopy, 5 mg/kg once daily (Archer et al., 2011). This supports published clinical findings of secondary infection in canine patients receiving cyclosporine at dosages as low as the doses recommended for use to treat canine atopic dermatitis.

The potential for the development of life‐threatening infections during cyclosporine therapy, in addition to the unpredictable bioavailability of oral cyclosporine, have prompted the establishment of individualized treatment strategies via pharmacodynamic monitoring in dogs. Utilizing a quantitative reverse transcription polymerase chain reaction (RT‐qPCR)–based assay, which measures the degree of immunosuppression at the molecular level in dogs receiving cyclosporine, dosing recommendations can be made with the goal of achieving acceptable immunosuppressive effects while minimizing the risk of secondary infections (Archer et al., 2011; Riggs et al., 2013).

When cyclosporine therapy needs to be abruptly discontinued when, for example, secondary infections are observed, it is expected that the patient's immune system will subsequently recover from the effects of the drug. However, there is no documented evidence in dogs regarding the time it takes for T‐cell function to recover following discontinuation of cyclosporine. This information would be of clinical value to veterinarians, especially clinicians managing patients that have developed serious secondary infections.

The objective of our study was to assess the time required for T‐cell function to return to pre‐treatment levels in healthy dogs after inducing immune suppression with cyclosporine, and subsequently abruptly withdrawing therapy. Based on previous work, it has been established that IL‐2 and IFN‐γ expression are markedly suppressed following 1 week of oral cyclosporine at immunosuppressive doses, and that cytokine gene expression can be determined using a standardized RT‐qPCR assay (Archer et al., 2011; Riggs et al., 2013). We hypothesized, based on experience with clinical samples in our laboratory, that cytokine expression would begin to recover within 2–3 days of discontinuing cyclosporine, and that full recovery to pre‐treatment values would occur within 1 week of cessation of drug therapy.

## 2 MATERIAL AND METHODS

All procedures were performed in accordance with the National Institutes of Health Guide for the Care and Use of Laboratory Animals, and study protocols were approved by the Institutional Animal Care and Use Committee. All animals used in this project were housed in the AAALAC accredited facilities.

### 2.1 Dogs

This project involved six healthy adult female Walker hounds. Prior to the study, each dog was deemed healthy by receiving a physical examination, complete blood count, serum biochemistry profile, urinalysis, faecal flotation and heartworm testing, with no significant abnormalities noted. The dogs were not exposed to any medications or vaccinations for at least one month prior to the start of the study.

### 2.2 Drug administration

Three mLs of blood was collected from each dog in a heparinized tube for baseline RT‐qPCR analysis for assessment of IL‐2 and IFN‐γ. Each dog then received oral cyclosporine (Atopica^®^ Novartis Animal Health, 10 mg/kg q12 hr) for 6 full days. All dogs were dosed on an empty stomach throughout the study. On Day 7, blood was once again collected in a heparinized tube from each dog, 2 hr after the morning cyclosporine dose was administered, for RT‐qPCR analysis. After this sample was collected, cyclosporine was discontinued. Each day after Day 7, for an additional 7 days, blood was collected in the morning from each dog in a heparinized tube for ongoing RT‐qPCR analysis.

### 2.3 Blood incubation and T‐cell activation

All blood samples (pre‐treatment samples, samples 2 hr after the last drug dose on Day 7, and daily samples for 7 days after cessation of cyclosporine) were activated prior to RNA extraction with 12.5 ng/mL of phorbol myristate acetate (PMA) (Sigma) and 0.8 μM of ionomycin (Sigma), as previously described (Riggs et al., 2013). All samples were then incubated for 5 hr at 37°C and 5% CO_2_.

### 2.4 RNA extraction and cytokine gene expression quantification

RNA was extracted using a previously published protocol (Riggs et al., 2013). Total RNA was isolated from 1 mL of heparinized whole blood using a QIAamp Whole Blood RNA Mini Kit (Qiagen, Cat. No. 52304). Genomic DNA was removed from the samples according to the manufacturer's instructions of an on‐column DNase (27.27 Kunitz units) treatment (Qiagen, Cat. No. 79254). Samples were then stored at −80°C until further analysis. The concentration and purity of the RNA were estimated by a Nanodrop ND‐1000 spectrophotometer using ND‐1000 V3.3.0 software (NanoDrop Technologies). A SuperScript™ III Platinum® SYBR® Green One‐Step kit with Rox as a reference dye (Invitrogen, Cat no. 11736‐059) was used to quantify expression of the genes of interest (IL‐2 and IFN‐γ) and the expression of the housekeeping gene GAPDH. Primers were based on GenBank sequences previously published by Kobayashi, Momoi, and Iwasaki (2007). All reactions were performed on a Stratagene Mx3500P Multiplex Quantitative PCR system (Agilent Technologies) integrated with MxPro software. The RT‐qPCR reaction was performed with a final volume of 20 µL containing a total of 30 ng of template RNA and 200 nM of each primer. The following thermal cycling parameters were used: 50°C for 3 min, 95°C for 5 min, then 40 cycles of 95°C for 15 s and 60°C for 30 s. Melting curve analysis comprised of the following parameters: 95°C for 15 s, 60°C for 1 min, after which the ramp speed was decreased from 1.667°C/sec to 0.01667°C/sec and data were collected continuously until it reached 95°C where the temperature was held for 30 s, and finally held at 60°C for 15 s. All samples were run in triplicate, whereas non‐template controls were run in duplicate. Delta cycle threshold (ΔCt) values were compared for pre‐treatment activated baseline samples, samples on Day 7 of cyclosporine therapy, and samples from each day of the recovery period after cyclosporine had been discontinued, where ΔCt = Ct_GOI_ − Ct_norm_, in which GOI is the gene of interest and norm is the reference gene. The 2^−∆∆Ct^ method was used to determine the relative change in expression using GAPDH as a reference gene, where ΔΔCt = (Ct_GOI_ − Ct_norm_)_treated_ − (Ct_GOI_ − Ct_norm_)_pre‐treatment_ (Livak & Schmittgen, 2001). Cytokine gene expression was also presented as a percentage compared with pre‐treatment activated baseline samples for samples collected on Day 7 of cyclosporine, as well as for samples collected during the recovery period, where pre‐treatment activated baseline samples represented 100% gene expression for IL‐2 and IFN‐γ. Relative expression was calculated using the formula: (2^−ΔΔCt^) × 100%.

### 2.5 Statistical analysis

The effect of sample time on each of IL‐2 and IFN‐γ ΔCt values was assessed by mixed‐model analysis using PROC MIXED, SAS for Windows 9.4 (SAS Institute, Inc.). Separate models were made for each cytokine. For each model, sample time was included as the fixed effect. The effect of repeated measures of each dog over time was accounted for using a repeated statement with an ante‐dependence correlation structure. If the effect of sample time was found to be significant, pairwise comparisons were made between the sample time 1 value (pre‐treatment activated baseline samples) and each of the values for sample time 2 through 9 (immediately following cessation of cyclosporine therapy as well as the 7 days in the recovery period) using Dunnett's adjustment for multiple comparisons of least squares means. The distribution of the conditional residuals was evaluated for each mixed model to ensure the assumptions of normality and homoscedasticity had been met for the statistical method. An alpha level of 0.05 was used to determine statistical significance.

## 3 RESULTS

### 3.1 IL‐2 and IFN‐γ analysis

**Figure 1:**
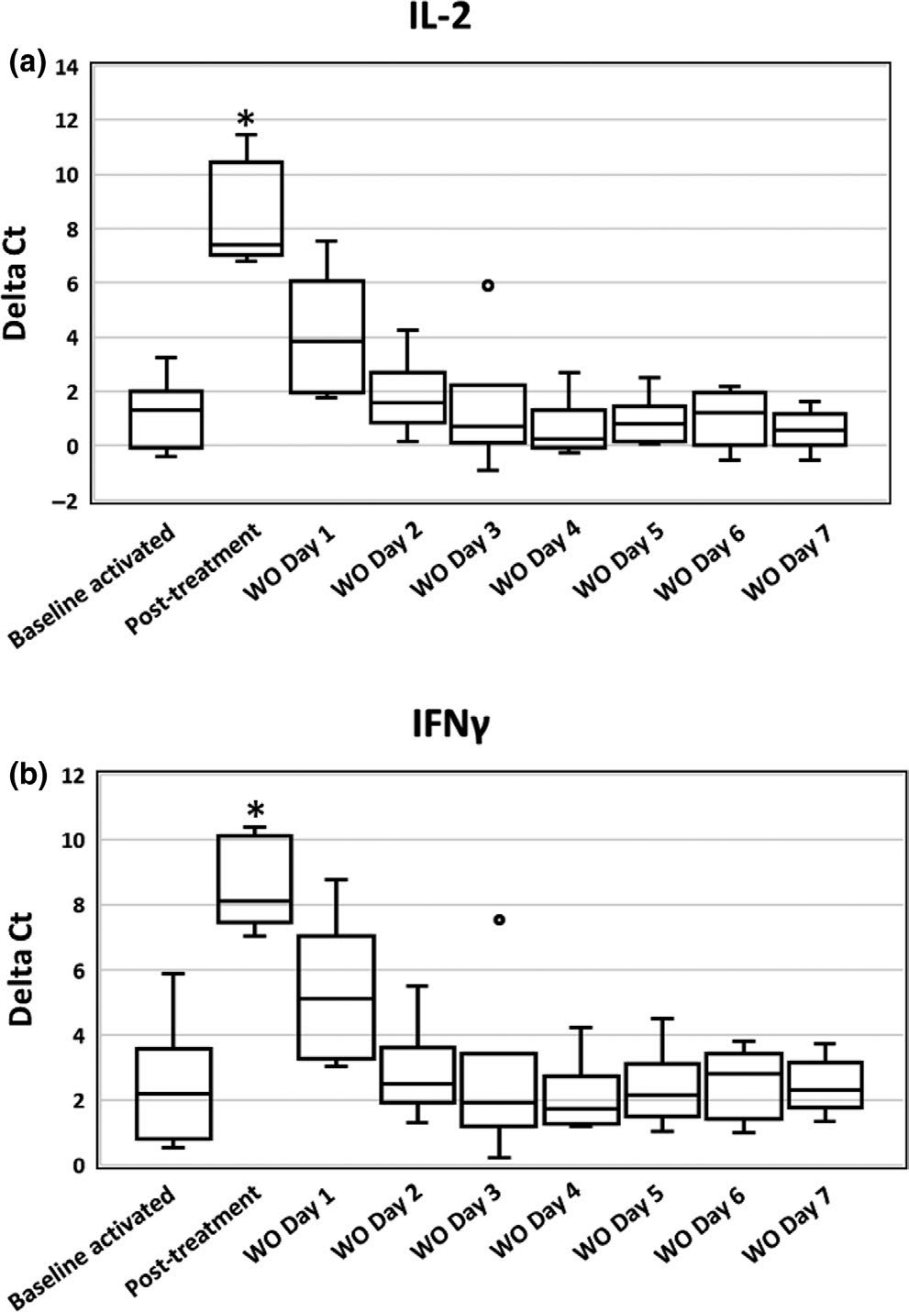
Box plots of activated whole‐blood IL‐2 (a) and IFN‐γ (b) RNA expression for six healthy Walker hounds. The effects of cyclosporine and the subsequent recovery of T‐cell cytokine RNA expression over 7 days following cessation of cyclosporine are presented. Expression is presented as ∆Ct values, where ΔCt = Ct_GOI_ − Ct_norm_, in which GOI is the gene of interest and norm is the reference gene. Increased ∆Ct numbers correspond to decreased cytokine RNA expression. The line within each box denotes the median, box edges represent the first and third quartiles, and whiskers extend to maximum and minimum values. ° Signifies outlier data points. Results on Day 7 of cyclosporine therapy (2 hr after administration of the last dose) were significantly different from untreated activated baseline samples (*p* < .004) as denoted with an asterisk (*). (IL‐2 = interleukin‐2, IFN‐γ = interferon‐gamma, WO = washout [recovery period])

**Figure 2:**
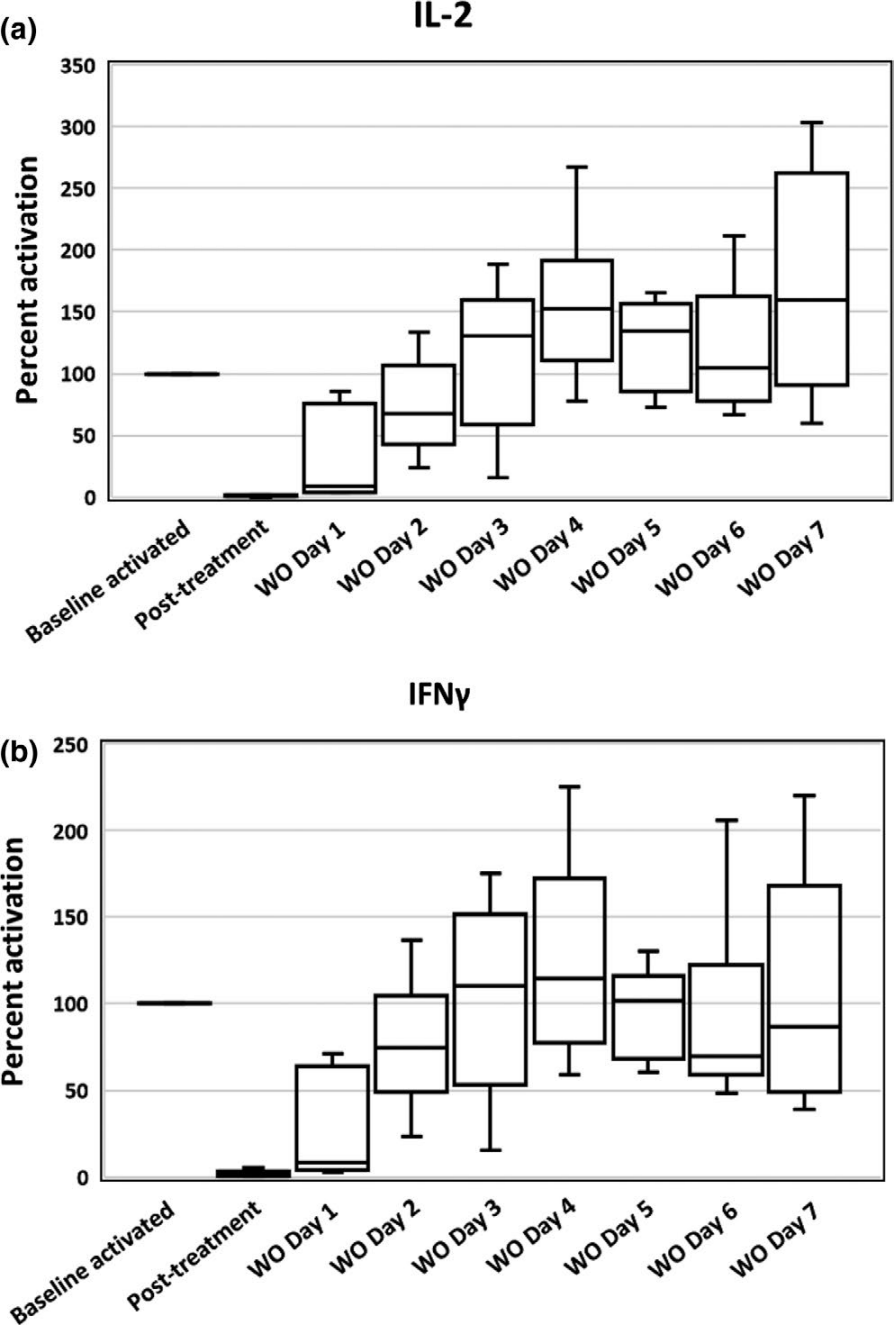
Box plots of activated whole‐blood IL‐2 (a) and IFN‐γ (b) RNA expression presented as a percentage of untreated activated baseline samples, in which the untreated activated baseline samples represent 100% cytokine production, from six healthy Walker hounds. The effects of cyclosporine and the subsequent recovery of T‐cell cytokine RNA expression over 7 days following cessation of cyclosporine are presented. The line within each box denotes the median, box edges represent the first and third quartiles, and whiskers extend to maximum and minimum values. Less than 5% of pre‐treatment values is clinically categorized as marked suppression (IL‐2 = interleukin‐2, IFN‐γ = interferon‐gamma, WO = washout [recovery period])

Quantitative reverse transcription polymerase chain reaction (RT‐qPCR) results are presented in Figures 1 and 2 for IL‐2 (a) and IFN‐γ (b). In Figure 1, ∆Ct values are presented. Increased ∆Ct numbers correspond to decreased cytokine RNA expression. Sample time was seen to have a significant effect on both IL‐2 (*p* = .008) and IFN‐γ (*p* = .007) ∆Ct values. When comparing the ∆Ct values of the pre‐treatment baseline activated samples to the samples collected on Day 7 of cyclosporine, there was a significant difference with IL‐2 (*p* = .002) and IFN‐γ (*p* = .004), indicating a significant suppression of cytokine expression following 1 week of cyclosporine. When comparing pre‐treatment baseline activated samples to all the daily samples during the recovery period after cessation of cyclosporine, a significant difference was not seen for either cytokine on any day (*p* > .0939). Statistical analysis was completed using the ∆Ct values.

Cytokine expression, presented as a percentage of pre‐treatment baseline activated samples, was also evaluated, and is shown in Figure 2. Pre‐treatment baseline activated samples represents 100% cytokine production. Median % of pre‐treatment baseline activated samples for day 7 of cyclosporine and the following 7 days are presented in Table 1. For samples collected on Day 7 of cyclosporine, median % (interquartile range) of pre‐treatment baseline activated samples was 0.9% (0.5) (corresponding to 99.1% suppression) for IL‐2 and 1% (1.7) (corresponding to 99% suppression) for IFN‐γ. Median % (interquartile range) of pre‐treatment baseline activated samples on Day 1 of the recovery period was 8.6% (52.9) for IL‐2 and 8% (43.3) for IFN‐γ. By Day 2, median % (interquartile range) of pre‐treatment baseline activated samples had increased to 67.4% (36.5) for IL‐2 and 74.3% (32.0) for IFN‐γ, and medians were greater than 100% for both cytokines by Day 3, suggesting full recovery. The Day 7 cyclosporine samples did show a marked suppression of cytokine expression following 7 days of drug administration, demonstrating that the assay worked in a manner consistent with previously published studies (Archer et al., 2011; Riggs et al., 2013).

**Table 1:** Median per cent (interquartile range) of pre‐treatment baseline activated samples

	Cyclosporine Day 7	Recovery Day 1	Recovery Day 2	Recovery Day 3	Recovery Day 4	Recovery Day 5	Recovery Day 6	Recovery Day 7
IL‐2	0.9% (0.5)	8.6% (52.9)	67.4% (36.5)	130.8% (64.8)	152.7% (35.5)	135% (49.7)	104% (54.2)	160% (116)
IFN‐γ	1% (1.7)	8% (43.3)	74.3% (32.0)	110.2% (64.9)	115% (67.7)	101% (35.2)	70% (25.4)	87% (80.2)

When individual dog ∆Ct values and cytokine expression as a percentage of pre‐treatment baseline activated samples were evaluated for IL‐2, two dogs returned to near pre‐treatment values (past within 20% of baseline values) by Day 2, two dogs returned to near pre‐treatment values by Day 3 and the final two dogs returned to near pre‐treatment values by Day 4 of the recovery period. When individual dog ∆Ct values and cytokine expression as a percentage of pre‐treatment baseline activated samples were evaluated for IFN‐γ, three dogs returned to near pre‐treatment values by Day 2, one dog returned to near pre‐treatment values by Day 3 and the final two dogs returned to near pre‐treatment values by Day 4 of the recovery period.

## 4 DISCUSSION

In this study, we utilized a RT‐qPCR based assay, validated in dogs, to explore the time taken for T‐cell cytokine production to recover after cessation of cyclosporine. Our study demonstrated that, following cessation of immunosuppressive dosages of cyclosporine, T‐cell expression of IL‐2 and IFN‐γ, when assessing ∆Ct values, was not statistically different from pre‐treatment values after the initial 24 hr of the drug recovery period in dogs.

Cyclosporine is used in the treatment of a number of immune‐mediated and inflammatory diseases in dogs, including IMHA, IMT, IMPA, atopy and anal furunculosis (Archer et al., 2014). Exposure to cyclosporine has been shown to markedly reduce activated T‐cell production of IL‐2 and IFN‐γ in dogs, both in vitro and in vivo (Archer et al., 2011; Fellman et al., 2011). The recommended oral starting dosage of cyclosporine for atopic dermatitis in dogs is 5 mg/kg once daily, and for systemic immune‐mediated disorders is approximately 5 mg/kg twice daily. We chose an oral cyclosporine dosage of 10 mg/kg twice daily, which is still within in the accepted dosage range for initial therapy in dogs (albeit at the highest end of the range) because we wanted to reliably induce immune suppression in all dogs in this study. The more common starting oral cyclosporine dosage of 5 mg/kg twice daily, based on our clinical experience with dogs receiving cyclosporine therapy, does not reliably induce immune suppression in all dogs.

In our study, statistical analysis was performed on the ∆Ct of both IL‐2 and IFN‐γ, comparing pre‐treatment baseline activated samples to all other samples. This method of analysis is consistent with other studies evaluating changes using a RT‐qPCR–based assay.

We also evaluated our results using the 2^−∆∆Ct^ method to generate a measure of degree of suppression of T‐cell cytokine expression, calculated as a percentage of the pre‐treatment activated baseline samples (Figure 2). Although the 2^−∆∆Ct^ method is not typically used for statistical analysis, it is often used clinically to compare the degree of cytokine suppression during treatment to a baseline pre‐treatment value. In clinical samples, per correspondence with the laboratory running the assay, % of pre‐treatment baseline activated samples between 0% and 5% (corresponding to a % suppression between 95% and 100%) is considered marked suppression, a % of pre‐treatment baseline activated samples between 5% and 20% is considered moderate high suppression, a % of pre‐treatment baseline activated samples between 20% and 50% is considered moderate suppression, a % of pre‐treatment baseline activated samples between 50% and 75% is considered low suppression, and a % of pre‐treatment baseline activated samples between 75% and 100% is considered minimal or no suppression. When the data from our study were evaluated using the 2^−∆∆Ct^ method, the median % of pre‐treatment baseline activated samples on Day 7 of cyclosporine administration for IL‐2 was 0.9%, and for IFN‐γ was 1%. This corresponds to the “marked suppression” category in clinical patients, which is the highest category of immune suppression that can be achieved. In the initial days of the recovery period following cessation of cyclosporine, there was a rapid return of T‐cell function as evidenced by a lessened amount of cytokine suppression, and return to full function (both ∆Ct values and % of pre‐treatment baseline activated samples returning to near baseline values for both cytokines) was seen by Day 4 of the recovery period for all dogs. There may have been a potential post‐recovery rebound effect, with the highest median % of pre‐treatment baseline activated samples for IL‐2 being 160% on Day 7 of the recovery period and 115% for IFN‐γ on Day 4 of the recovery period, although the low animal numbers and day‐to‐day and dog‐to‐dog variability in results preclude any definitive conclusions.

Our study used all female dogs of a single breed, and used healthy dogs rather than dogs with inflammatory or immune‐mediated diseases. It is possible that differences exist between genders or breeds, or between healthy and diseased dogs. Additional studies in dogs of varying breeds, sexes and health statuses are indicated, to determine if similar findings exist in a range of clinic patients with different naturally occurring diseases. Our study was also underpowered for detecting subtle effects, as evidenced by the fact that, whereas there was a marked and statistically significant difference between results attained while the dogs were receiving cyclosporine compared with both pre‐treatment and recovery period values, we did not detect significant day‐by‐day differences in results over the immediate recovery period. Typically, six to eight dogs are used for standard pharmacokinetic and pharmacodynamic studies, as described by Riviere, and published by our research group for previous pharmacodynamic studies (Fellman et al., 2016; Haraschak et al., 2014; Riviere, 1999). However, although within the acceptable number of dogs for a pharmacodynamic study, the use of six dogs may not have provided sufficient power to detect clinically important changes in ∆Ct values in the first few days of the post‐cyclosporine recovery period, and care must therefore be taken extrapolating data to larger populations.

In conclusion, T‐cell expression of IL‐2 and IFN‐γ in healthy dogs will begin to return to pre‐treatment values within 24 hr of cessation of cyclosporine, but it may take several more days for cytokine expression to consistently return to pre‐treatment values. This timeframe should be kept in mind when stopping cyclosporine therapy in clinical patients, especially those that need abrupt cessation of therapy because of the development of secondary infections.

## CONFLICT OF INTEREST

Drs. Archer, Mackin and Thomason are affiliated with the Mississippi State University Pharmacodynamic Laboratory, which provides the assay evaluated in this study as a commercial service to veterinarians.

## ETHICAL STATEMENT

The authors confirm that the ethical policies of the journal, as noted on the journal's author guidelines page, have been adhered to and the appropriate ethical review committee approval has been received. The US National Research Council's guidelines for the Care and Use of Laboratory Animals were followed.
